# Enhanced vision language framework for medical report generation using agent-based retrieval

**DOI:** 10.3389/fdgth.2026.1989756

**Published:** 2026-10-19

**Authors:** Anandhu Saji, Fousia M. Shamsudeen, Chinnu Jacob, Cristina Popirlan, Claudiu-Ionut Popirlan, D. Jude Hemanth

**Affiliations:** 1 Centre for AI, TKM College of Engineering, Kollam, India; 2 Department of MCA, TKM College of Engineering, Kollam, India; 3 Faculty of Sciences, Department of Computer Science, University of Craiova, Craiova, Romania; 4 Department of Electronics and Communication Engineering, Karunya Institute of Technology and Sciences, Coimbatore, India

**Keywords:** agent-based retrieval, chest X-ray, medical imaging, radiology report generation, retrieval-augmented generation, vision-language models

## Abstract

**Introduction:**

Automated chest X-ray report generation has the potential to reduce the workload of radiologists by combining deep learning and natural language processing. However, existing vision-language models (VLMs), including R2Gen, may generate incorrect or unsupported statements when relevant contextual information is insufficiently captured. This study proposes a modular framework to improve automated report generation through inference-time retrieval and language model-based refinement.

**Methods:**

Given an input chest X-ray image, R2Gen generates a preliminary report. Relevant contextual information is retrieved using image-derived and textual representations through a FAISS-based similarity retrieval mechanism. The retrieved textual information is combined with the preliminary report and provided to the Zephyr 7B language model to generate the final report. The framework is evaluated on the IU X-Ray and CheXpert Plus datasets using BLEU, ROUGE-L, METEOR, and token-overlap-based precision, recall, and F1-score. The retrieval and refinement stages operate at inference time without requiring additional retraining or fine-tuning of the underlying R2Gen model.

**Results:**

Experimental results demonstrate improved performance over the R2Gen baseline across the reported evaluation metrics on the evaluated datasets, indicating greater similarity between the generated reports and the reference reports.

**Discussion:**

The findings suggest that integrating similarity-based retrieval with language model-based refinement can improve the reported text-generation performance of automated chest X-ray reporting systems. The modular framework offers an inference-time approach that avoids additional training of the underlying report generation model.

## 1 Introduction

The Chest X-rays (CXRs) are the most frequently performed imaging tests in clinical practice due to their wide availability, cost-effectiveness, and low radiation doses. CXRs comprise approximately 40% of the 3.6 billion diagnostic X-ray examinations performed worldwide each year (1, 2). Physicians obtain CXRs for diverse purposes, including diagnosing disease, monitoring longitudinal disease progression, and verifying the placement of medical devices, among others. Writing radiology reports is a tedious and labor-intensive process, requiring trained specialists to conduct in-depth analyses of chest radiographs and create detailed reports of their findings. This process is also inherently restricted by a variety of human limitations, including the experience of the radiologist and availability of medical support staff. Therefore, automatically generating free-text radiology reports from chest radiographs has immense clinical value. Radiology report generation, which aims to automatically generate a free-text description for a clinical radiograph (e.g., chest X-ray), has emerged as a prominent research direction in artificial intelligence (AI) and clinical medicine. AI-powered radiology report generation can significantly enhance work- flow automation, improve reporting efficiency, and standardize healthcare practices (3). However, existing methodologies often struggle with challenges such as factual inconsistencies, hallucinations, and the inability to incorporate multimodal data effectively. Practically, a significant challenge of radiology report generation is that radiology reports are long narratives consisting of multiple sentences. A radiology report generally consists of a section of findings, which describes medical observations, including both normal and abnormal features, as well as an impression or concluding remark summarizing the most prominent observations. Conventional image captioning approaches (4, 5) may be insufficient for radiology report generation, as these methods are designed for brief scene descriptions rather than comprehensive medical analyses. Accurate and contextually appropriate report generation is an important requirement in the computer aided analysis of medical images.

Vision language models have shown considerable potential for generating radiology reports from medical images. However, the information available during report generation is generally based on the representations learned during model development. Incorporating relevant information from previously available reports can provide additional context and may help the model generate reports that are better aligned with the available clinical information. In this work, a modular framework is developed for chest X ray report generation by combining preliminary report generation, retrieval during inference, and language model based report refinement. The framework first uses R2Gen to generate a preliminary report from the input chest X ray. Relevant contextual information is then retrieved from an external radiology report repository using image derived and textual representations. The retrieved textual context, together with the preliminary report, is subsequently provided to a separate Zephyr 7B based refinement stage to generate the final report. FAISS is used to support similarity based retrieval from the report repository.

Recent research has shown growing interest in retrieval based approaches for radiology report generation. In this context, the proposed framework focuses on a modular workflow that brings together preliminary report generation, contextual retrieval, and report refinement within the inference process. An important practical aspect of the framework is that the retrieval and refinement process does not require additional retraining or fine tuning of the underlying R2Gen model. The retrieval repository can therefore be extended or updated independently, with the corresponding retrieval index updated when required, while retaining the pretrained R2Gen component.

The framework is evaluated on the IU X Ray and CheXpert Plus datasets using BLEU, ROUGE L, METEOR, and token overlap based Precision, Recall, and F1 measures. The results are compared with the R2Gen baseline under the experimental configuration used in this study. The key contributions of the paper are as follows:

- Developed a modular retrieval assisted framework for chest X ray report generation by extending R2Gen with inference time retrieval and Zephyr 7B based report refinement.
- Integrated FAISS based similarity retrieval to identify relevant visual and textual contextual information from an external radiology report repository.
- Introduced an inference time retrieval and refinement strategy that incorporates additional contextual information without requiring retraining or fine tuning of the underlying R2Gen model.
- Evaluated the framework on the IU X Ray and CheXpert Plus datasets using BLEU, ROUGE L, METEOR, and token overlap based Precision, Recall, and F1 measures, demonstrating improved performance over the R2Gen baseline.

Rest of this paper is organized as follows. Section 2 presents Previous works. Section 3 describes the Proposed Methodology, Section 4 presents proposed framework Section 5 presents the experimental results include performance analyisis and Sections 6, 7 gives ablation study and comparison with state of art methods and Section 8 gives limitation and future scope and Section 9 concludes.

## 2 Previous works

Medical report generation has evolved significantly over the years, incorporating various deep learning techniques to enhance accuracy, coherence, and reliability. Given the complexity of radiology reports, numerous methodologies have been explored to automate this process while addressing the inherent challenges of missing, incorrect, or hallucinatory statements. Despite advancements in artificial intelligence (AI) and deep learning, the generation of medical reports remains a challenging task due to the intricate nature of medical imaging data, the necessity for precise diagnostic terminology, and the risk of hallucinations in AI-generated content. This section reviews existing approaches, their strengths, and their limitations, providing a foundation for further improvements in automated radiology report generation. Early approaches to radiology report generation relied on deep learning architectures such as Convolutional Neural Networks (6, 7). These models aimed to extract key information from medical images and translate it into coherent and contextually appropriate text. However, despite their innovative designs, these models often produced incomplete, inaccurate, or misleading statements, limiting their effectiveness in clinical settings. Studies have shown that even state-of-the-art models tend to generate reports containing missing or false references when evaluated by trained radiologists (8). CNN based approaches have been widely utilized for their ability to extract hierarchical visual features from X-ray images, which serve as critical inputs for medical report generation. Jing et al. (9) introduced an LSTM based approach where disease prediction was treated as an intermediate step before report generation. This methodology aimed to enhance diagnostic relevance by ensuring that gen erated text was aligned with the predicted medical conditions.

Transformer-based architectures, which have revolutionized natural language processing, have also been explored for radiology report generation. Chen et al. (10) showed that Transformer models improve contextual accuracy in multimodal report generation, while Wang et al. (11) enhanced this by pre-training a ViT with masked autoencoders on high-resolution X-rays for better feature extraction and report consistency. The emergence of Large Language Models (LLMs) has significantly influenced the field of radiology report generation. Traditional LLMs such as BERT (12) introduced bidirectional contextual understanding, greatly improving text comprehension and synthesis. Meta’s LLaMA (13) expanded on this concept by optimizing computational efficiency while maintaining strong performance in natural language generation. OpenAI’s GPT series, particularly GPT-3 (14), set new benchmarks in natural language processing, demonstrating remarkable capabilities in medical text synthesis and contextual understanding. The ability of these models to process vast amounts of textual data has made them invaluable for medical applications. Domain-specific LLMs have been developed to address the unique challenges associated with medical text generation. R2GenGPT (15) integrates both image and textual data using LLaMA2-7B (16), enhancing the accuracy and contextual awareness of generated reports. Additionally, MedVersa (17) extends these capabilities by supporting multimodal inputs, allowing for real-time adaptability and enhancing the precision of medical reporting.

One of the most significant advancements in medical report generation is the integration of Retrieval-Augmented Generation (RAG) models, which have been employed to mitigate factual inaccuracies in AI-generated reports (18–20). RAG models retrieve relevant medical knowledge before generating reports, thereby improving factual consistency and contextual relevance. A recent scoping review by Suganthi et al. (21) analyzed 45 RAG-based studies in radiology and reported that RAG consistently improves report generation and question answering performance. However, the review also highlighted that hallucinations, evaluation inconsistencies, and limited clinical validation remain significant challenges. Liu et al. (22) proposed Factual Serialization Enhancement, a two stage framework that first learns clinically grounded representations through factuality guided contrastive learning and subsequently retrieves similar historical cases as factual evidence for report generation. The retrieved evidence is incorporated into the generation process through a cross modal fusion mechanism.

Jia et al. (23) proposed Semantic Feedback Based RAG for Radiology Report Generation, referred to as RAGSemRad. The method introduces a fine grained semantic retrieval module that clusters report sentences according to semantic information and uses a pretrained medical vision language model to retrieve relevant semantic sentences. It further introduces a semantic assessment module that uses paired image label data to provide semantic supervision during training. Despite these advantages, conventional RAG approaches may retrieve information that is redundant or less relevant to the input, which can affect the quality of the generated report. To address these limitations, recent studies have explored Agentic RAG frameworks that employ multiple intelligent agents for dynamic retrieval and reasoning. Wong et al. (24) proposed a multi-agent conversational radiology system that combines DenseNet-121, Gemini 1.5 Pro, and a ChromaDB-based RAG module with a feedback agent to continuously improve report generation through previously verified clinical cases. Recent studies have also explored knowledge-enhanced and memory-augmented approaches to radiology report generation. Allaberdiev et al. (25, 26) investigated these directions through two complementary approaches. The first study proposed a self-critical multisource knowledge reasoning framework that incorporates multiple knowledge sources with large language models for radiology report generation, while the second introduced ChestXGen, a dynamic memory-augmented vision-language transformer with context-aware gating for incorporating contextual information during report generation.

These studies demonstrate that retrieval based radiology report generation has progressed beyond simple nearest neighbour retrieval toward semantically guided retrieval and clinically informed evidence integration. Building upon these advancements, the present work adopts a different modular organization. Instead of developing a new retrieval representation or introducing additional clinical label supervision, the proposed framework uses R2Gen to generate a preliminary report, performs retrieval during inference, and subsequently uses a separate Zephyr 7B based language model to refine the preliminary report using the retrieved textual context.

## 3 Methodology

The proposed methodology, shown in Figure 1, extends the R2Gen framework with a retrieval and report refinement stage for chest X ray report generation. The study uses the IU X Ray and CheXpert Plus datasets, where the available X ray images and associated reports are used for constructing the retrieval repository and evaluating the framework. For a given input X ray image, R2Gen first processes the image through its visual encoder and report generation network to produce a preliminary report. In the implemented pipeline, the chest X-ray image is the primary input to the report-generation process; no separate clinical history or other patient-provided non-visual information was supplied as an independent input to the generation stage. This preliminary report, together with image derived information, is then used in the retrieval stage to identify relevant reports from the repository using FAISS based similarity search. The retrieved report information is subsequently provided along with the preliminary R2Gen report to the Zephyr 7B language model, which generates the refined report. The Zephyr 7B refinement stage receives the preliminary R2Gen-generated report together with the retrieved textual report information through a fixed generation prompt. Image-derived information is incorporated during the preceding retrieval stage. The overall process consists of three stages: preliminary report generation, contextual information retrieval, and report refinement. In this work, the term “agent” refers to a functionally specialized module within a predefined processing pipeline rather than an autonomous multi-agent reasoning system. The Retrieval Agent performs the retrieval operation, while the Report Generation Agent performs the subsequent report refinement using Zephyr 7B. The framework thus provides a modular mechanism for incorporating additional contextual information during inference without requiring further fine tuning of the underlying R2Gen model.

**Figure 1:**
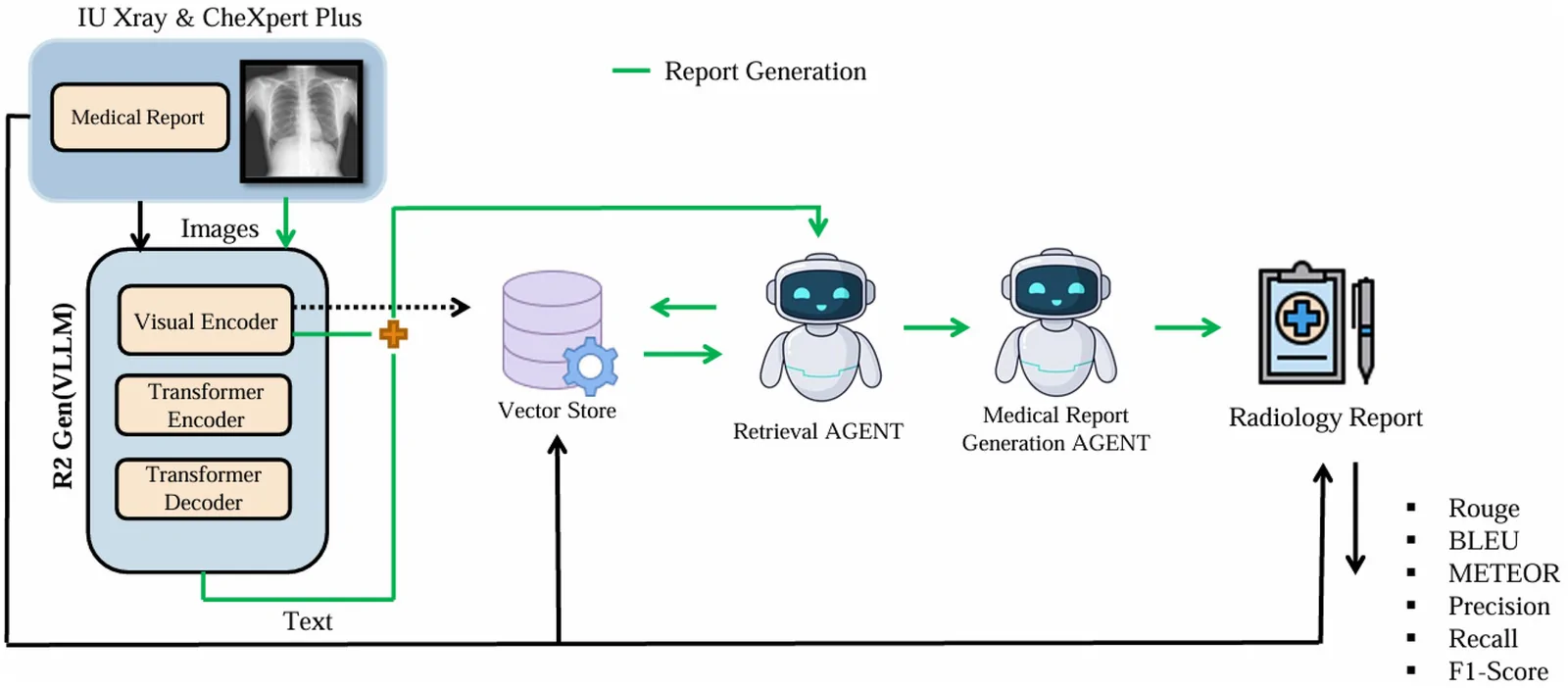
Proposed framework.

An important characteristic of the proposed implementation is that retrieval is performed during inference and does not require additional fine tuning of the underlying R2Gen report generation model. Consequently, the retrieval repository can be modified or extended while retaining the pretrained R2Gen component, subject to updating the corresponding retrieval index when required. This provides a modular mechanism for incorporating additional contextual information without retraining the core report generation model.

### 3.1 Datasets and evaluation metrics

We assess our model’s performance using three datasets: IU-XRay and CheXpert Plus. A brief overview of each dataset is provided below.

- IU-XRay (27) commonly used X-ray image datasets for medical report generation. It includes 7,470 chest X-Ray images and 3,955 radiology reports, each comprising four sections: Indication, Comparison, Findings, and Impression. In our work, we partition the dataset in an 80:20 ratio, allocating 80% for building the vector database and the remaining 20% for testing.
- CheXpert Plus (28) is a large well structured medical dataset, containing 223,000 X-ray image, report pairs from 64,700 patients. The dataset includes annotations for 14 chest conditions and patient data. In our study, we use the “Findings” section as the ground truth and split the dataset in an 80:20 ratio allocating 80% for vector database creation and 20% for testing.

For each dataset, the data were divided into an 80% retrieval portion and a 20% evaluation portion. The retrieval portion was used to construct the FAISS database and was not used for additional training or fine tuning of the R2Gen model. During inference, the images in the evaluation portion were treated as query cases, and relevant information was retrieved from the corresponding retrieval database. The ground truth report of each evaluation case was used only for calculating the evaluation metrics and was not provided to the report generation or refinement stage. The retrieval databases for IU X Ray and CheXpert Plus were constructed independently and were not combined. The original data preparation maintained patient level separation, with multiple views from the same patient retained within the same partition.

For evaluating the quality of radiology report generation, we employ several widely used metrics: ROUGE, BLEU, METEOR, Precision, Recall, and F1-score. ROUGE (Recall-Oriented Understudy for Gisting Evaluation) measures the overlap between the generated and reference reports, with a focus on recall. BLEU (Bilingual Evaluation Understudy) assesses the precision of n-gram matches between the generated and reference reports, helping to evaluate the quality of generated sentences. METEOR complements BLEU by considering unigram precision and recall while also accounting for stemming and synonym matching, making it more sensitive to linguistic variations. In addition, Precision, Recall, and F1-score are used to measure the overlap between the generated and reference reports. Precision indicates the proportion of generated tokens that match the reference report, Recall measures the proportion of reference tokens captured by the generated report, and F1-score provides a combined measure of Precision and Recall. These metrics collectively provide information about the linguistic quality and content overlap between the generated and reference reports. These metrics evaluate the agreement between the generated and reference reports under the adopted automatic evaluation protocol. Since these measures are sensitive to textual similarity, improvements in the reported scores are interpreted within the scope of automatic report-generation evaluation rather than as direct evidence of clinical correctness.

### 3.2 Experimental setup

In our experiments, chest X-ray images are directly passed to the model to extract the visual feature and the text output. The R2Gen component was initialized using a pretrained R2Gen checkpoint trained on IU X Ray. No additional training or fine tuning of R2Gen was performed using the retrieval or evaluation portions of the present experiment. The pretrained checkpoint therefore served as the initial model for preliminary report generation and visual feature extraction, while the retrieval database was used as a non parametric source of contextual information during inference. Our work utilizes both the IU-Xray and CheXpert Plus datasets for evaluating the report generation capability of the proposed method. All experiments are conducted on a system equipped with an NVIDIA GeForce RTX 4060 GPU (8GB VRAM). The Zephyr 7B refinement stage is invoked through the Hugging Face Hub interface. It receives the preliminary R2Gen-generated report together with the retrieved textual report information through the report-generation prompt. No additional fine-tuning of the R2Gen model was performed during the retrieval or evaluation stages.

## 4 Proposed framework

### 4.1 Vector database construction

Let $D=\{(I_{i},R_{i}){\}}_{i=1}^{N}$ denote the datasets, where each sample consists of a chest X-ray image $I_{i}\in R^{H\times W}$ and its corresponding radiology report $R_{i}$.

#### 4.1.1 R2Gen visual feature extraction

We employ the **R2Gen** model, a vision-language architecture designed for medical report generation. The visual feature extractor in the implemented R2Gen architecture is a CNN-based ResNet-101 encoder, which maps the input chest X-ray image to a dense visual feature representation.The model comprises:

- A *visual extractor* $f_{v}\;:\;R^{H\times W}\to R^{d}$, which maps an input image $I_{i}$ to a dense visual feature representation $v_{i}\in R^{d}$.
- A *language decoder* $f_{\text{dec}}\;:\;R^{d}\to Y$, used later for report generation.

For each image $I_{i}$, the visual representation is obtained as expressed in Equation (1):

$$v_{i}=f_{v}(I_{i})\in R^{d}$$ (1)

#### 4.1.2 Radiology report text embedding

Each corresponding report $R_{i}$ is converted into a fixed-dimensional semantic embedding using a pre-trained sentence embedding model $f_{t}\;:\;T\to R^{k}$, specifically the sentence transformers/all-MiniLM-L6-v2 model expressed in Equation (2):

$$u_{i}=f_{t}(R_{i})\in R^{k}$$ (2)

where $u_{i}\in R^{k}$ captures the semantic content of the radiology report.

#### 4.1.3 Index construction in FAISS

A **vector database** V is constructed using FAISS for efficient similarity search. For each sample $(I_{i},R_{i})\in D$, we store:

- The *visual feature vector* $v_{i}$ as the searchable vector in the FAISS index.
- The corresponding *textual embedding* $u_{i}$ alongside the indexed visual feature for semantic comparison during inference.

Thus, each entry in the FAISS index is represented as:

$${\text{Entry}}_{i}=\left(v_{i},u_{i}\right)$$ (3)

The final vector store is:

$$V={\left\{{\text{Entry}}_{i}\right\}}_{i=1}^{N}$$ (4)

This structure enables multimodal retrieval by storing both the visual feature vector $v_{i}$ and its corresponding textual embedding $u_{i}$. During inference, visual similarity is computed using the indexed feature vectors $v_{i}$, while the associated textual embeddings $u_{i}$ are used for semantic comparison, thereby enhancing downstream radiology report generation.

### 4.2 Radiology report generation via R2Gen with agent-based retrieval

Let $I_{q}\in R^{H\times W}$ be the query chest X-ray image for which a diagnostic report needs to be generated. The proposed agent-based system involves two core agents: the *Retrieval Agent* and the *Report Generation Agent*.

#### 4.2.1 Stage 1: visual encoding and initial report generation

The query image is passed through the R2Gen model to extract a visual feature vector and an initial report as expressed in Equations (5) and (6):

$$v_{q}=f_{v}(I_{q})\in R^{d}$$ (5)

$${\hat{R}}_{q}=f_{\text{R2Gen}}(v_{q})$$ (6)

Here, $f_{v}$ is the image encoder and $f_{\text{R2Gen}}$ is the full R2Gen model that generates a preliminary report ${\hat{R}}_{q}$ from visual features.

#### 4.2.2 Stage 2: embedding the generated report

The initial report ${\hat{R}}_{q}$ is embedded using a sentence transformer model $f_{t}$ to obtain the textual query vector as given in Equation (7).

$$u_{q}=f_{t}({\hat{R}}_{q})\in R^{k}$$ (7)

#### 4.2.3 Stage 3: retrieval by retrieval agent

The **Retrieval Agent** queries the FAISS vector database $V=\{(v_{i},{\text{metadata}}_{i}=\{u_{i}\}){\}}_{i=1}^{N}$ using both the image and textual embeddings:

- $v_{q}$ is used for visual similarity search.
- $u_{q}$ is used for semantic similarity search in the metadata.

The similarity is computed using a joint scoring function S as given in Equation (8):

$${\text{Score}}_{i}=S(v_{q},v_{i},u_{q},u_{i})=\text{sim}(v_{q},v_{i})+\text{sim}(u_{q},u_{i})$$ (8)

where sim(⋅,⋅) is the cosine similarity function

The top-K most similar cases $\{(v_{i^{*}},u_{i^{*}}){\}}_{i^{*}\in I_{K}}$ are retrieved as given in Equation (9).

$$R=\text{TopK}\left({\left\{{\text{Score}}_{i}\right\}}_{i=1}^{N}\right)$$ (9)

The retrieved database entry is associated with its corresponding report information in the repository. The selected report information is recovered from the associated metadata and provided as textual context to the Zephyr 7B refinement stage.

#### 4.2.4 Stage 4: final report generation by report generation agent

The **Report Generation Agent** takes the retrieved relevant information R to generate a content-enriched report as expressed in Equation (10):

$${\tilde{R}}_{q}=f_{\mathrm{Zephyr}}({\hat{R}}_{q},C_{q};P)$$ (10)

where ${\hat{R}}_{q}$ denotes the preliminary report generated by R2Gen, $C_{q}$ denotes the retrieved textual report information, and P denotes the predefined generation prompt used for report refinement. The query image influences the refinement process through the preceding R2Gen and retrieval stages. The final report ${\tilde{R}}_{q}$ is generated by refining the preliminary report ${\hat{R}}_{q}$ using the retrieved textual report information $C_{q}$. The overall procedure of the proposed framework is summarized in Algorithm 1.

Algorithm 1 **Radiology Report Generation using R2Gen with Agents.**

**Input:** Set of chest X-ray images $\{I_{i}{\}}_{i=1}^{N}$ and corresponding reports $\{R_{i}{\}}_{i=1}^{N}$

**Output:** Enriched radiology report for query image $I_{q}$

1: **Phase 1: Vector Database Construction**

2: **for each** image-report pair $\left(I_{i},R_{i}\right)$ **do**

3: Extract image feature vector using R2Gen image encoder: $\;\;\;\;v_{i}=f_{v}(I_{i})$

4:  Generate optional report: ${\hat{R}}_{i}=f_{R2\mathrm{Gen}}(v_{i})$

5:  Compute text embedding of report: $u_{i}=f_{t}(R_{i})$

6:  Store $\left(v_{i},u_{i}\right)$ in FAISS vector database

7: **end for**

8: Construct vector database $V=\{(v_{i},u_{i}){\}}_{i=1}^{N}$

9: **Phase 2: Retrieval and Report Refinement**

10: Extract query image features: $v_{q}=f_{v}(I_{q})$

11: Generate preliminary report: ${\hat{R}}_{q}=f_{R2\mathrm{Gen}}(v_{q})$

12: Compute text embedding: $u_{q}=f_{t}({\hat{R}}_{q})$

13: **for each** $\left(v_{i},u_{i}\right)$ in database

14:  Compute similarity score:

15:  $\mathrm{Scor}e_{i}=\mathrm{sim}(v_{q},v_{i})+\mathrm{sim}(u_{q},u_{i})$

16: **end for**

17: Retrieve top-K similar entries based on $\mathrm{Scor}e_{i}$

18: Use retrieved cases to generate enriched final report

19: **Return:** Generated report

## 5 Experimental results

The proposed R2Gen-based agent-assisted retrieval framework was rigorously evaluated on two prominent radiology report generation datasets CheXpert Plus and IU X-Rays using standard evaluation metrics as discussed above. These metrics jointly assess linguistic overlap, semantic accuracy, and clinical relevance between the generated reports and corresponding ground truth references.

On the CheXpert Plus dataset, the model achieved a B4 score of 0.2085, reflecting its ability to capture n-gram level patterns and produce fluent language closely matching the reference reports. The RL score of 0.3622 and METEOR score of 0.3917 indicate a high level of lexical and semantic overlap, respectively. Furthermore, the model obtained a Precision of 0.5847, Recall of 0.3857, and F1-score of 0.4546, indicating the corresponding performance of the generated reports according to these evaluation measures.

On the IU X-Rays dataset, the performance metrics were significantly higher: B4 reached 0.3939, R-L was 0.5070, and METEOR stood at 0.5382, indicating higher agreement between the generated and reference reports according to the reported evaluation measures. Additionally, it achieved a Precision of 0.5997, Recall of 0.6976, and F1-score of 0.6338, further reflecting the reported performance of the generated reports under the adopted evaluation measures. These results indicate improved performance according to the evaluation measures adopted in this study.

To supplement the quantitative results, a qualitative comparison was performed between the generated and the reference ground truth report, as illustrated in Figure 2. The visual analysis reveals that the model-generated report captures crucial diagnostic elements such as: Absence of acute pathology, Clear lung fields and absence of effusions or consolidations, Normal cardiac silhouette and mediastinal contours and Intact bony structures without evidence of fracture or destructive lesion. These observations show agreement between the generated report and the corresponding content in the ground truth, illustrating the ability of the framework to generate coherent and contextually appropriate radiological summaries.

**Figure 2:**
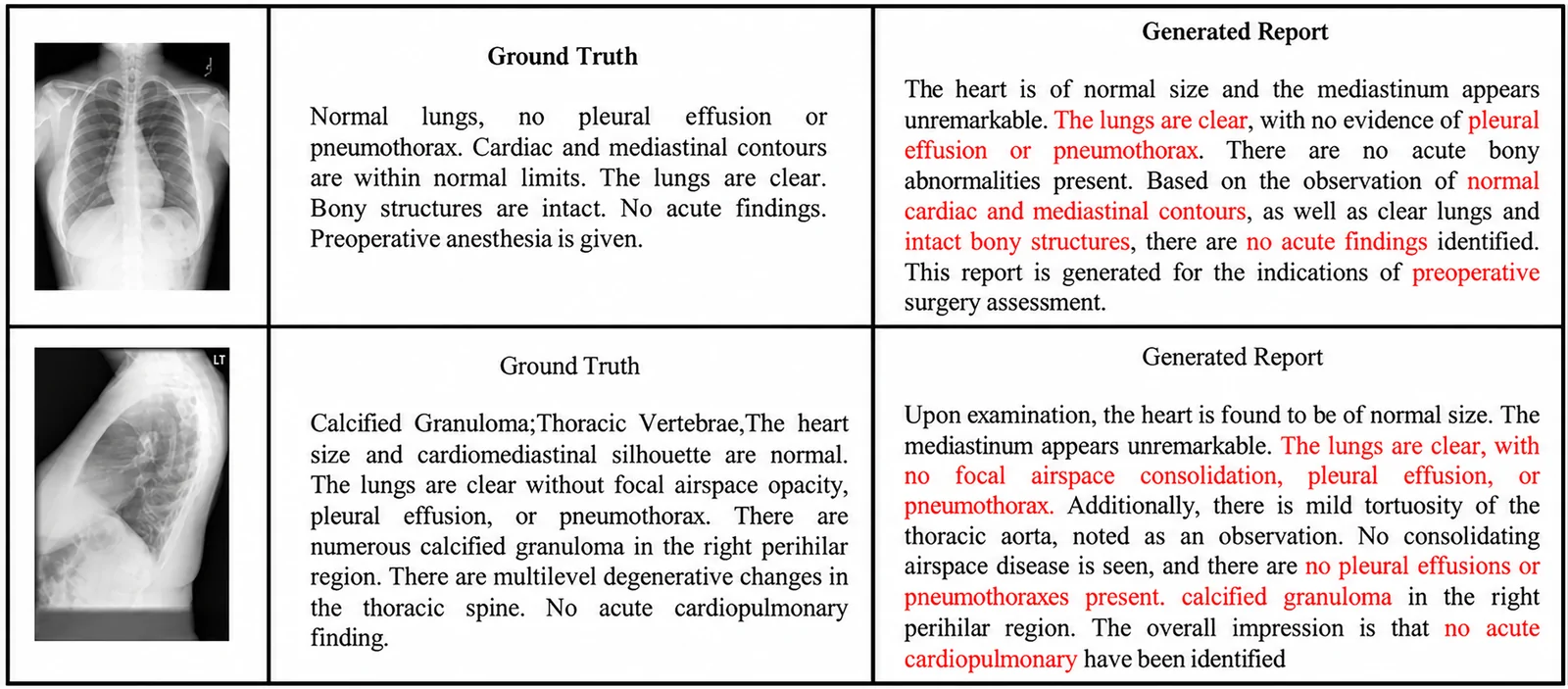
Qualitative comparison of the generated report and the corresponding ground-truth report. Common text is highlighted in red.

The integration of the retrieval mechanism provides additional information from previously available radiology reports during the report-generation process. Unlike the baseline R2Gen model, the proposed framework uses retrieved report information together with the preliminary R2Gen report in the refinement stage. This additional information supports the generation of coherent radiology reports, with the framework showing improved performance across the reported evaluation metrics.

## 6 Ablation study

An ablation study was conducted to compare the R2Gen baseline implemented in the present study with the complete proposed retrieval-and-refinement framework. The results across the two benchmark datasets, CheXpert Plus and IU X-Ray, are summarized in Table 1. The baseline R2Gen model achieved a BLEU-4 score of 0.081 on CheXpert Plus and 0.165 on IU X-Ray, while the proposed framework achieved BLEU-4 scores of 0.2085 and 0.3939, respectively. Similar differences were observed for ROUGE-L and METEOR on both datasets. These results indicate improved performance of the complete proposed framework according to the evaluation measures adopted in this study.

**Table 1:** Ablation study comparing the R2Gen baseline implemented in the present study with the proposed framework on the CheXpert Plus and IU X-Ray datasets using standard NLG evaluation metrics (BLEU-4, ROUGE-L, METEOR, Precision, Recall, and F1-score).

Model	CheXpert Plus						IU X-Rays					
	B4	R.L	M	P	R.s	F1	B4	R.L	M	P	R	F1
R2Gen	0.081	0.246	0.318	0.318	0.240	0.257	0.165	0.371	0.187	–	–	–
Proposed method	**0.2085**	**0.3622**	**0.3917**	**0.5847**	**0.3857**	**0.4546**	**0.3939**	**0.5070**	**0.5382**	**0.5997**	**0.6976**	**0.6338**

Bold values indicate the results obtained by the proposed method in the present study.

## 7 Comparison with state-of-the-art-methods

In the realm of automatic radiology report generation, balancing linguistic fluency with clinical accuracy has long been a challenge. Traditional encoder decoder models like R2Gen have laid the foundation for this task by introducing transformer based mechanisms tailored to the unique demands of medical report generation. However, despite their early success, such models often suffer from limited context awareness, poor factual grounding, and a static understanding of patient data. Most recent approaches have focused heavily on fine-tuning large language models (LLMs) to overcome these limitations. While effective to some extent, fine-tuning comes with significant computational overhead and suffers from rigidity new knowledge cannot be easily integrated without retraining the entire model.

The proposed framework extends R2Gen by incorporating an inference-time retrieval stage followed by Zephyr 7B-based report refinement. The retrieved report information is subsequently used together with the preliminary R2Gen report during the refinement stage. The framework therefore combines retrieval and language-model-based refinement within the inference workflow, while retaining the underlying R2Gen model without additional fine-tuning.

To substantiate these claims, we conducted comprehensive experiments on two benchmark datasets: CheXpert Plus and IU X-Rays. The results are tabulated in Table 2. On the CheXpert Plus dataset, the proposed method achieved a BLEU-4 score of 0.2085, ROUGE-L of 0.3622, METEOR of 0.3917, precision of 0.5847, recall of 0.3857, and F1-score of 0.4546. On the IU X-Ray dataset, the proposed framework achieved BLEU-4, ROUGE-L, METEOR, precision, recall, and F1-score values of 0.3939, 0.5070, 0.5382, 0.5997, 0.6976, and 0.6338, respectively. These results demonstrate improved performance across the reported evaluation metrics on both datasets.

**Table 2:** Performance comparison with previously reported radiology report generation results on the CheXpert Plus and IU X-Ray datasets.

Dataset	Algorithm	B4	R-L	M	P	R	F1
CheXpert Plus	R2Gen (29)	0.081	0.246	0.113	0.31	0.200	0.181
	R2GenGPT (29)	0.101	0.266	0.145	0.315	0.244	0.260
	R2GenCSR (29)	0.100	0.265	0.146	0.315	0.247	0.259
	VLC1 (29)	0.080	0.247	0.114	0.341	0.175	0.163
	MambaXray-VL-L (29)	0.112	0.276	0.157	0.377	0.319	0.335
	**Proposed method**	**0.2085**	**0.3622**	**0.3917**	**0.5847**	**0.3857**	**0.4546**
IU X-Rays	R2Gen (10)	0.165	0.371	0.187	–	–	–
	R2GenCMN (30)	0.170	0.375	0.191	–	–	–
	MambaXray-VL-L (29)	0.185	0.371	0.216	–	–	–
	METTransformer (31)	0.172	0.380	0.192	–	–	–
	Token-Mixer (32)	0.190	0.402	0.208	–	–	–
	BootstrappingLLM (33)	0.184	0.390	0.208	–	–	–
	R2GenGPT (15)	0.173	0.377	0.211	–	–	–
	R2GenCSR (34)	0.206	0.401	0.215	–	–	–
	**Proposed method**	**0.3939**	**0.5070**	**0.5382**	**0.5997**	**0.6976**	**0.6338**

Results are reported using BLEU-4 (B4), ROUGE-L (R-L), METEOR (M), Precision (P), Recall (R), and F1-score (F1), with the proposed work results highlighted in bold.

The results for the comparison methods in Table 2 are taken from their corresponding publications, whereas the results of the proposed method are obtained in the present study.The proposed framework reports higher numerical values than the corresponding published results shown in Table 2. These values are presented for comparison with previously reported methods. On the CheXpert Plus dataset, the proposed method achieved a BLEU-4 score of 0.2085 and an F1-score of 0.4546, compared with 0.112 and 0.335, respectively, for MambaXray-VL-L. Similarly, on the IU X-Ray dataset, the proposed framework achieved BLEU-4, ROUGE-L, and METEOR scores of 0.3939, 0.5070, and 0.5382, respectively, compared with 0.206, 0.401, and 0.215 reported for R2GenCSR. The proposed method also achieved precision, recall, and F1-score values of 0.5997, 0.6976, and 0.6338, respectively, on the IU X-Ray dataset.

On the IU X-Ray dataset, the proposed method achieved a BLEU-4 score of 0.3939, while the published R2Gen value is 0.165. Higher values were also observed for ROUGE-L and METEOR, with the proposed method achieving scores of 0.5070 and 0.5382, respectively, while the published R2Gen values are 0.371 and 0.187. These results indicate improved agreement between the generated and reference reports according to the adopted evaluation metrics.

The proposed framework is characterized by a modular retrieval and report-refinement process in which relevant contextual information is retrieved at generation time and subsequently used to refine the preliminary report. The framework retrieves relevant information through vector-based retrieval and uses the retrieved textual context together with the preliminary report during the report-refinement stage. This provides additional contextual information that supports the generation of more coherent reports.

In conclusion, the results provide empirical evidence that integrating an agent-based retrieval mechanism with the R2Gen architecture improves its performance across the reported report-generation metrics. The framework incorporates retrieval during inference without additional fine-tuning of the underlying R2Gen model and demonstrates improved performance over the R2Gen baseline under the evaluation settings used in this study. The comparison with previously published results is provided as a reference to existing methods, since the underlying evaluation protocols may not be identical. These findings highlight the potential of retrieval-augmented report generation mechanisms for medical AI and provide opportunities for further investigation of retrieval-based approaches in clinical decision support applications.

## 8 Discussion

The results indicate that adding inference-time retrieval and report refinement to the R2Gen pipeline improves the reported performance under the evaluation setting used in this study. The framework first generates a preliminary report using R2Gen and then uses retrieved textual information during the refinement stage to provide additional context for the final report. The ablation results compare the complete retrieval-and-refinement framework with the R2Gen baseline and therefore reflect the combined effect of the proposed workflow.

The evaluation uses BLEU, ROUGE-L, METEOR, Precision, Recall, and F1-score to compare the generated reports with their corresponding reference reports. These measures provide information about the similarity and content agreement between the generated and reference reports. The results are therefore interpreted within the scope of the automatic evaluation measures used in this study.

The retrieval stage relies on information available in the report repository, and the characteristics of this repository can influence the retrieved context provided during report refinement. The current evaluation focuses on chest X-ray report generation using the IU X-Ray and CheXpert Plus datasets. Evaluation across additional datasets and medical imaging modalities would provide further evidence regarding the applicability of the framework. Similarly, more advanced vision-language or multimodal models could be incorporated to examine how different visual and language representations affect the retrieval and report-refinement process.

## 9 Conclusion

This study presented a modular framework for chest X ray report generation by extending the established R2Gen model with retrieval during inference and a separate report refinement stage using Zephyr 7B. The framework first generates a preliminary report using R2Gen, retrieves relevant contextual information from the report repository, and then uses the retrieved information together with the preliminary report to generate the final report. The experimental results obtained on the IU X Ray and CheXpert Plus datasets show improved performance over the R2Gen baseline across the reported evaluation measures, indicating better alignment between the generated and reference reports under the experimental settings used in this study.

The main contribution of the work is the modular organization of established report generation, retrieval, and language model components rather than the introduction of a new retrieval algorithm. An important practical aspect of the framework is that retrieval is performed during inference without additional fine tuning of the underlying R2Gen model. The retrieval repository can therefore be extended or updated, with the corresponding retrieval index updated when required, while retaining the pretrained R2Gen component.

The present study is focused on chest X ray report generation, and the evaluation is based on automatic report generation and token overlap measures. Further investigation using dedicated clinical factuality measures and evaluation on other medical imaging modalities would be useful to assess the broader applicability of the framework. Future work can also explore more clinically informed retrieval strategies and additional validation of the generated reports.

## Code availability

The code supporting this study is available at: https://github.com/AnandhuSaji007/Agentic-Rag-for-Xray-Medical-Report-Generation.

## Data Availability

The original contributions presented in the study are included in the article/Supplementary Material, further inquiries can be directed to the corresponding author.
